# Concurrent validity of the combined HRV/ACC sensor and physical activity diary when monitoring physical activity in university students during free-living days

**DOI:** 10.3389/fpubh.2022.950074

**Published:** 2022-09-08

**Authors:** Haochong Liu, Qian Li, Yiting Li, Yubo Wang, Yaling Huang, Dapeng Bao, Haoyang Liu, Yixiong Cui

**Affiliations:** ^1^Sports Coaching College, Beijing Sport University, Beijing, China; ^2^School of Sport Medicine and Physical Therapy, Beijing Sport University, Beijing, China; ^3^China Institute of Sport and Health Science, Beijing Sport University, Beijing, China; ^4^Institute of Sports Strategy, Beijing Sport University, Beijing, China; ^5^AI Sports Engineering Lab, School of Sports Engineering, Beijing Sport University, Beijing, China

**Keywords:** physical activity, energy expenditure, validity, intensity, free-living

## Abstract

The purpose of this research was to determine if the scientific research device combined heart rate variability combined with an acceleration sensor (Firstbeat Bodyguard 2, BG2) was valid and reliable for time spent in different intensity zones in free-living. A total of 55 healthy participants performed 48-h physical activity (PA) monitoring with BG2, ActiGraph GT3X+ (GT3X+), and completed Bouchard Physical Activity Diary (Bouchard) every night. In the available studies, GT3X+ is considered the gold standard scientific research device for PA monitor. We compared BG2 and Bouchard with GT3X+ by difference, correlation, and agreement of PA and energy expenditure (EE) in free-living. The results showed that BG2 estimated PA more accurately than Bouchard, with a modest correlation (*r* > 0.49), strong agreement (τ > 0.29), and they had the lowest limits of agreement when estimating moderate to vigorous physical activity (MVPA). The EE estimated by Bouchard was the highest among the three methods, and the correlation and agreement between the three methods were high. Our findings showed that the BG2 is valid and reliable for estimating time spent in different intensity zones in free-living, especially in MVPA.

## Introduction

Physical activity (PA) that produces physical energy expenditure (total energy expenditure or TEE) is defined as any movement that results in a physiological response. Regular PA reduces the risk of mortality and morbidity, regardless of other alterations in lifestyle (1). It is recommended that adults between the ages of 18 and 64 years engage in 150–300 mins of moderate-intensity aerobic activity per week or 75–150 min of high-intensity aerobic activity per week in accordance with the 2020 WHO Guidelines (2). Although energy expenditure (EE) is a significant factor in PA and energy balance, maintaining a specific volume of moderate-to-vigorous physical exercise activity (MVPA) is more important, which is regarded as one of the recommended approaches for preventing cardiovascular risk (3). In comparison, people tend to ignore intensity when completing the recommended amount of exercise (4), which might lead to the phenomenon where people reach the recommended EE under continuous low-intensity PA. Therefore, MVPA in our daily activities should be emphasized considering the benefits of cardiorespiratory fitness.

Currently, various wearable microtechnologies such as accelerometer (ACC), pedometer, and heart rate monitor have been common in PA monitoring, among which ACC-based sensors have been widely utilized within scientific research (5). Specifically, the Actigraph GT3X+ accelerometer is recognized as a reliable tool for adults under free-living conditions to achieve such purpose (6). However, because of their signal detection and data processing techniques, they inevitably result in bias in estimated EE and time of different activity intensities (7, 8). Due to the limitation of wearing position, ACCs often ignore lower limb-based movements, such as climbing stairs, pedaling, etc. Therefore, many studies have also developed corresponding algorithms based on the type of PA (9). Previous studies assessing free-living intensity-specific PA and sedentary (SD) had observed variations in ACC's inter-instrument reliability across intensity categories. The greatest variation has been shown with coefficients of variation (CV) for SD (CV = 10.5%), MPA (CV = 13.5%), and VPA (CV = 12.3%) and very VPA (CV = 18.2%) (10). Trost et al. classified 12 different physical activities into seven categories (lying down, sitting, standing, walking, running, basketball, and dancing), using machine learning techniques, and the classification accuracy for the hip worn accelerometer was 91.0% ± 3.1%. However, it offers modest accuracy in dancing (64.1% for hip and 69.4% for wrist), and approximately 25% of the dancing trials were misclassified as standing plus activities (11). Such results indicate that a single monitoring system might not be able to distinguish between dance and activities requiring upright posture and arm movement (e.g., sweeping the floor). Therefore, future studies should explore the viability of multiple monitor systems.

Firstbeat Bodyguard 2 (BG2, Firstbeat Technologies Ltd, Jyväskylä, Finland) is a body physiology modeling sensor based on the HRV and tri-axial ACC data, calibrated for personal information such as date of birth, gender, height, weight, maximum HR, and minimum HR. BG2 is able to deeply differentiate between mental stress and PA by combining techniques to further improve the analytical recognition ability of PA. Thus, combining technologies should theoretically improve the accuracy of EE estimates and calculate activity time at different intensities with greater precision (12). The BG2 is a commercially available device that provide reliable R–R interval and movement data and could be used in both free-living and lab scenarios. Preliminary studies have demonstrated the advantages of integrating physiological measures, such as heart rate variability combined with an acceleration sensor (HRV/ACC) in the estimation of TEE (13). Nonetheless, no available study has focused on the accuracy of the time spent in different intensities by BG2.

Besides, many studies used activity diaries conveniently for PA estimation. Despite this, diary estimates tend to be biased compared with the real life. Activity diaries are less effective for intensity estimation because of the inherent method limitations and participants' comprehension biases (14).

Based on the above-mentioned rationale, this study aimed to (i) compare the intensity-specific PA time spent estimated by BG2 and Bouchard with that of GT3X+ to evaluate the concurrent validity of BG2; (ii) compare the estimated EE by three methods relatively. It was hypothesized that combining HRV/ACC data exhibited higher consistency on time spent in different intensity zones. As the study takes into account the free-living scene, it is expected that the findings of the study will inform researchers with a more appropriate alternative to monitor PA in generally population.

## Methods and measures

### Participants

This study recruited 102 healthy adults (58 men and 62 women) who signed a written informed consent before experiment. The inclusion criteria before experiment were as follows: (i) all participants with good sleep (PSQI score < 5); (ii) have no injury history affecting PA in the past 6 months; (iii) do not participate in another biomedical study during the experiment; and (iv) avoid consuming alcohol or caffeinated foods, or any drugs that affect HRV were included in the study. And the exclusion criteria after experiment were as follows: (i) failure to complete the daily Bouchard (more details are shown in study protocol); (ii) the percentage of valid data from GT3X+ was <80%; (iii) the error percentage of BG2 data was >25% according to the suggestion of manufacturer. A total of 55 physically active participants (28 men: age 22.9 ± 2.7 years, height 177.9 ± 6.2 cm, weight 74.7 ± 9.8 kg, and BMI 23.5 ± 2.2 kg/m^2^; 27 women: age 22.2 ± 2.5 years, height 164.1 ± 5.7 cm, weight 58.2 ± 8.0 kg, and BMI 21.6 ± 2.6 kg/m^2^) were finally included in this study. The Experimental Ethics Committee approved the study for Sports Science at Beijing Sport University (Registration number 2022056H). Participants were informed of the procedures and purpose of the study.

### Study protocol

**Figure 1:**
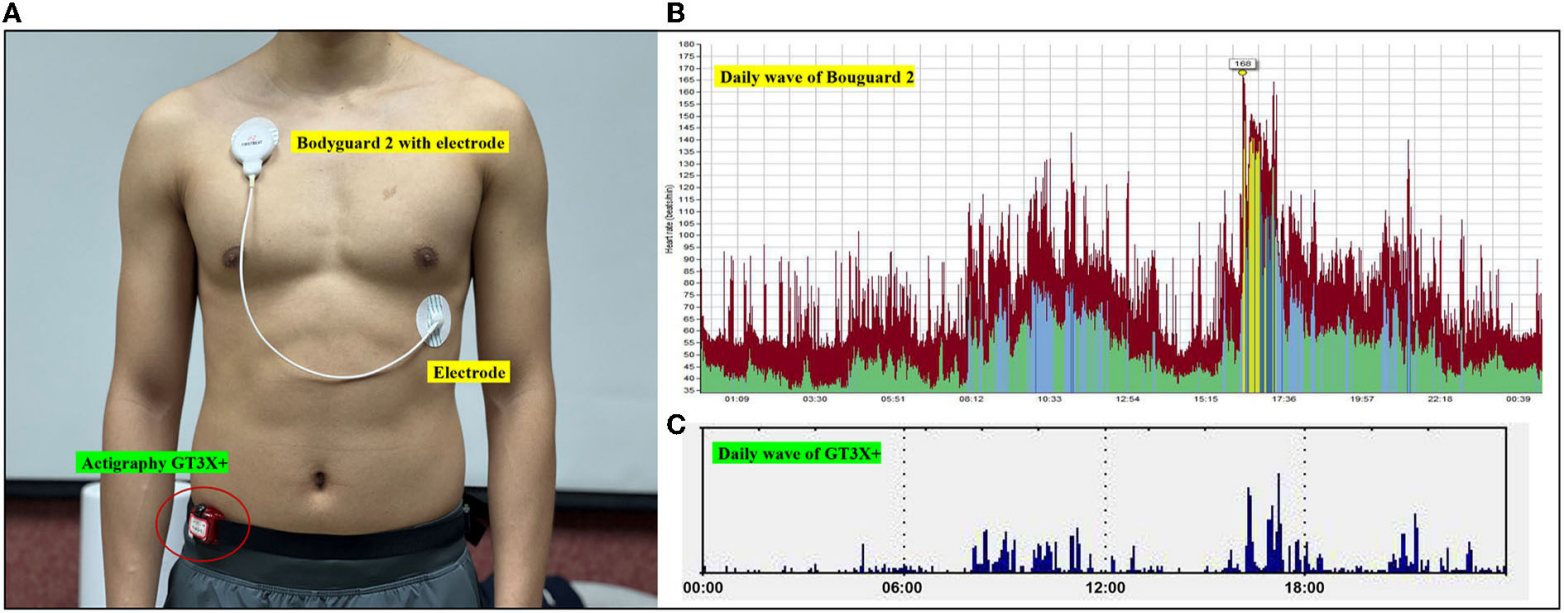
**(A)** Photographs of participants wearing BG2 and GT3X+ during free-living. **(B)** Screenshot of the software program used to acquire PA parameters for BG2. **(C)** Screenshot of the software program used to acquire PA parameters for from GT3X+.

In December 2021, 48-h PA monitoring has completed for this study, winter (average air temperature: −4 to 5°C and relative humidity: 18–33%). During free-living testing, participants wore GT3X+ and BG2. Figure 1 shows how the devices should be worn and a screenshot of the data. The participants were required to wear the devices throughout the day, except during bathing, swimming, and sleep. Devices were modeled according to the manufacturer's instructions and configured for the individual's age/date of birth, sex, and name before testing. Moreover, participants need to finish the Bouchard nightly with recall and auxiliary records.

**Accelerometer** (GT3X+) is a type of tri-axial ACC that is commonly used by researchers (15). SD and PA may be measured based on activity counts in an epoch with a set of intensity thresholds, i.e., activity counts classified by time intervals (epoch length) (9). Participants wore a GT3X+ (sampling rate 30 Hz) attached to an elastic waistband around the side of the dominant hip (16). Subjects were monitored for 48 consecutive hours of PA data using a GT3X+, and the data were initially collated and graded using its companion software, Actilife 6.13. At first, Troiano Adult's (2008) criteria were chosen as the basis for classifying PA, where 0–99 counts/min is considered SD, 100 to 2,019 counts/min is LPA, and ≥2,020 counts/min is MVPA, using this criterion to calculate SD time, LPA time, MVPA time and the proportion of each component, as shown in Table 1. Second, Freemason VM3 Combination (2011) formula is chosen to set the PA energy (PAEE).

**Table 1:** Correlation and agreement of PA parameters in BG2 and Bouchard compared with GT3X+.

		**Pearson (*r*)**	**Kendall's tau-b (τ)**	**ICC (95%CI)**
SD (min/d)	Bouchard vs. GT3X+	0.48	0.29**	0.33 (0.08 to 0.54)
	BG2 vs. GT3X+	0.49	0.34**	0.08 (−0.06 to 0.26)
LPA (min/d)	Bouchard vs. GT3X+	−0.02	0.06	−0.01 (−0.28 to 0.25)
	BG2 vs. GT3X+	0.53	0.37**	0.06 (−0.05 to 0.21)
MVPA (min/d)	Bouchard vs. GT3X+	0.40	0.22*	0.16 (−0.07 to 0.39)
	BG2 vs. GT3X+	0.55	0.29**	0.48 (0.25 to 0.66)
TEE (kcal/d)	Bouchard vs. GT3X+	0.90	0.71**	0.87 (0.74 to 0.93)
	BG2 vs. GT3X+	0.84	0.64**	0.83 (0.73 to 0.90)
PAEE (kcal/d)	Bouchard vs. GT3X+	0.79	0.50**	0.71 (0.48 to 0.84)
	BG2 vs. GT3X+	0.52	0.34**	0.55 (0.34 to 0.71)

*P*-values in Kendall's tau-b indicates statistical significance (^*^P < 0.05 and ^**^P < 0.01).

**Combined HRV/ACC sensors** (BG2) could monitor beat-to-beat heart rate that targeted for long-term monitoring of HRV and PA (17). It is attached to the body through two electrodes placed under the right clavicle and on the left lower lateral area of the ribcage. Following the recording, registered data are transferred to a computer and analyzed using dedicated software (Firstbeat Sports; v4.5.0.2.) (18).

The subjects wore a BG2 to monitor their PA for 48 h. The data were initially collated and graded using Firstbeat SPORTS software and analyzed and calculated according to the following criteria to obtain indicators such as time in PA at different intensities and TEE calculated by HRmax and activity level (0–10). Different intensity zones are divided as follows: 30–49% light intensity; 50–69% moderate intensity; and ≥70% high intensity to vigorous intensity (19).

**Physical activity diary** (Bouchard) used in this study and it is one of the most commonly used diaries and involves recording. Bouchard consisting of 96 15-min blocks per day (24 h) during two consecutive days (48 h) (20). Participants were asked to record the main activity in each 15-min block and rate the action on a scale of intensity (1–9, 1 being the lowest and 9 the highest intensity). Each numeric activity code refers to a specific energy cost and converted to a metabolic ratio of expended energy (MET). Total diary EE was calculated as the amount of time in each period multiplied by the correspondent MET and the estimated basal metabolic ratio (BMR). BMR was calculated using the prediction formulas by sex, age, weight, and height. The formula is EE (kcal) for a particular type of PA = EE standard for that type of activity [kcal/(kg−15 min)] × total period (number of sessions) for that type of activity; TEE (kcal /d) = ∑ 2 days of EE for every kind of PA/2. The participants complete the Bouchard every night before going to sleep. Classification of exercise intensity intervals based on Bouchard that Categorical values 1, 2 are considered SD; Categorical values 3, 4 are considered LPA; and Categorical values 5–9 are considered MVPA.

**Quality control:** The enumerators in this study were trained in a uniform and rigorous manner, and double-entry was used. All the types of electronic equipment were purchased from the same batch. Questionnaires and diaries were checked for omissions at the time of collection, and if any were completed on the spot. Subjects were asked to check their equipment while wearing the sensor, and record their activity every hour in the group *via* instant messaging to improve the accuracy of the recording to some extent.

### Data processing and analysis

The TEE was automatically exported in BG2, and TEE was calculated according to the formula in Bouchard. PAEE in BG2 and Bouchard were estimated by assuming a fixed percentage for the thermic effect of food (10% of TEE) and a standard resting EE (REE) of 25 kcal/kg/day [PAEE = (TEE × 0.9) – REE] (21). PAEE was automatically exported in GT3X+. Yet, the TEE in GT3X+ was also calculated by the same prediction equations.

### Statistical analysis

Data were tested for normality using mean ± SD for descriptive analysis. The correlations between three methods by the Pearson correlation coefficient were classified as having no relationship Strength of the correlation was interpreted as 0.0–0.1 trivial; 0.1–0.3 small; 0.3–0.5 moderate; 0.5–0.7 large; 0.7–0.9 very large and 0.9–1.0 nearly perfect (22). The differences between the three methods by using paired *t*-test. Cohen's *d* was used as effect size statistics for the paired *t*-test and was calculated and interpreted according to the following thresholds: 0.2 trivial; 0.6 small; 1.2 moderate; 2.0 large; 4.0 very large; and ≥4.0 extremely large (22). The agreement between three methods by Kendall's tau-b and ICC. Kendall's tau-b were classified as 0.10–0.19 weak; 0.20–0.29 moderate; 0.30 or above strong. The ICC is a value between 0 and 1, where values below 0.5 indicate poor reliability, between 0.5 and 0.75 moderate reliability, between 0.75 and 0.9 good reliability, and any value above 0.9 indicates excellent reliability (23).

The statistical analysis was performed using IBM SPSS Statistics 26.0. The significance level is defined as *P* < 0.05. The Bland–Altman plots using the Medcalc 19.6.4 compared the consistency of the three methods were generated (24).

## Results

**Figure 2:**
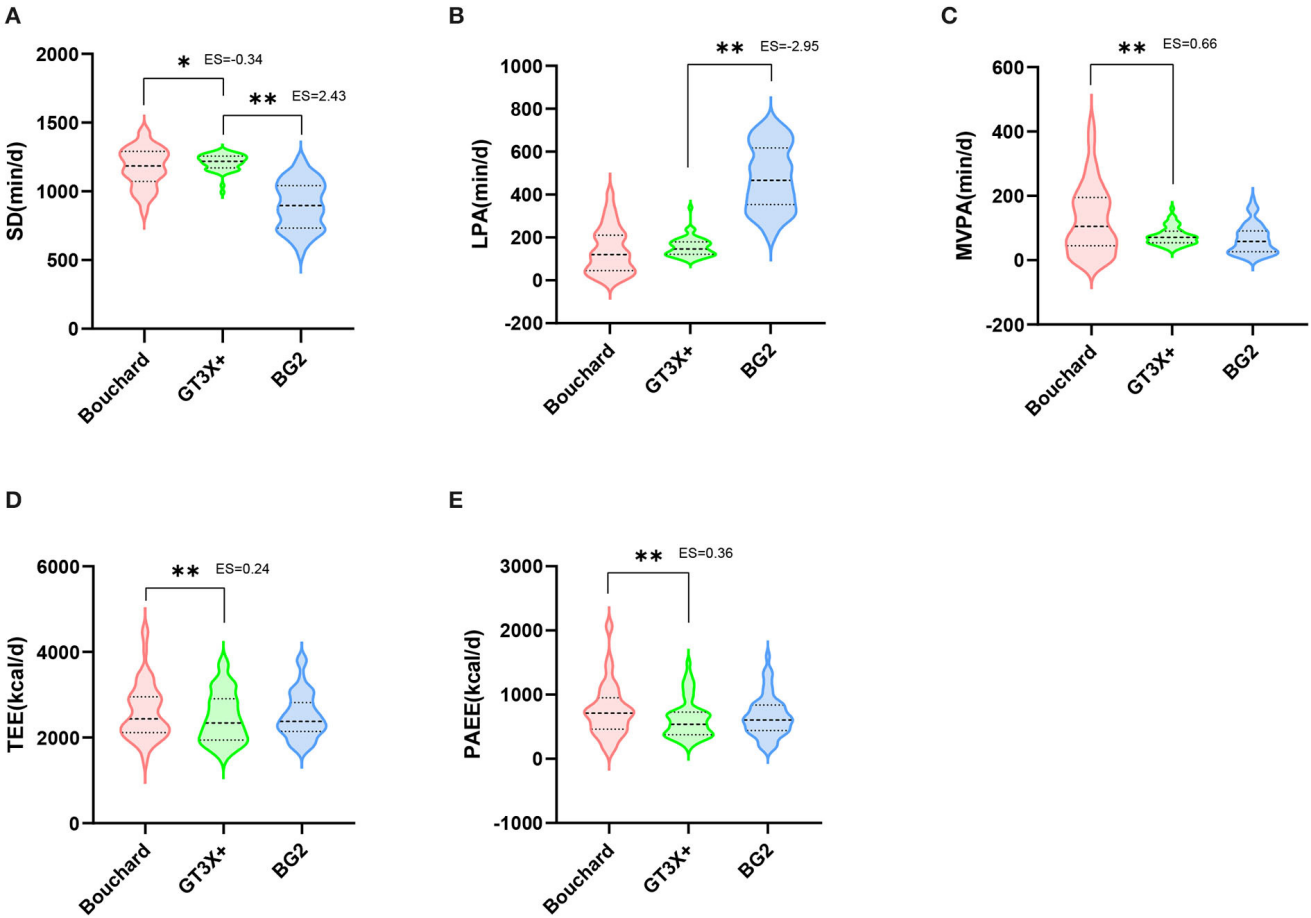
The differences of SD, LPA, MVPA, TEE, and PAEE during the free-living by three methods. The thicker dashed line represents the median, and the thinner dashed line represents the inter quartile range. Two-tailed paired *t*-test *P*-values indicate statistical significance (*P < 0.05 and **P < 0.01).

**Figure 3:**
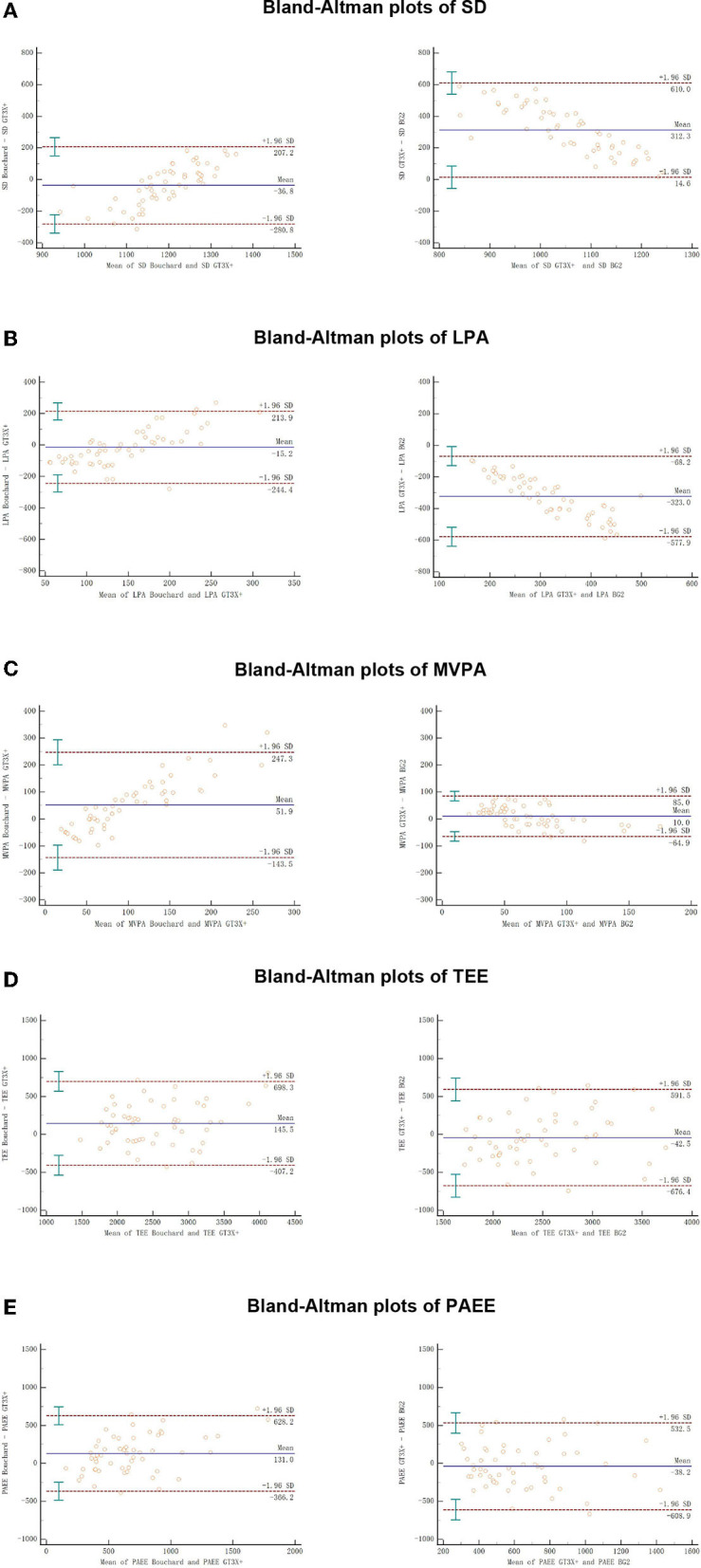
The Bland–Altman plots for SD, LPA, MVPA, TEE, and PAEE during the free-living by three methods. Solid lines show the mean difference between methods, and dotted lines show the 95% CI of the limits of agreement (Mean ± 1.96 SD); error bars are 95% CIs.

The differences of time spent in different intensity zones and EE during the free-living by three methods were shown in Figure 2. The correlation and agreement of PA parameters between Bouchard, GT3X+ and BG2 were show in Table 1. The Bland–Altman plots for time spent in different intensity zones and EE during the free-living by three methods were shown in Figure 3.

### Differences of PA in free-living between three methods

The BG2 and Bouchard were significantly lower than GT3X+ in SD estimation (*P* < 0.01, ES > −0.34). BG2 was significantly higher than GT3X+ in LPA (*P* < 0.01, ES = −2.95), while Bouchard showed no significant difference in this regard. For MVPA, Bouchard was significantly higher than that measured by GT3X+ (*P* < 0.01, ES = 0.66), while BG2 has no significant difference with GT3X+. Similarly, BG2 showed no significant difference from GT3X+ in TEE and PAEE estimation, whereas Bouchard was significantly higher than that observed in GT3X+ (*P* < 0.01, ES > 0.24).

### Correlation and agreement of PA in free-living between three methods

For the time spent in different intensity zones, the correlation of BG2 is higher than Bouchard in both LPA and MVPA. BG2 has a moderate correlation compared with the GT3X+ (*r* > 0.49), whereas Bouchard has a trivial correlation (*r* = −0.02) in LPA. Similarly, the agreement of BG2 with GT3X+ (τ = 0.37, ICC = 0.06) is clearly higher than Bouchard in LPA (τ = 0.06, ICC = −0.01). A similar pattern was observed in MVPA that BG2 (*r* = 0.55, τ = 0.29, ICC = 0.48) showed relatively better correlation, agreement and ICC values than Bouchard (*r* = 0.40, τ = 0.22, ICC = 0.16).

## Discussion

This study is the first to examine the accuracy of the BG2 and Bouchard for estimating PA in healthy adults during free-living. The current results indicate that estimating time spent in different intensity zones estimated by BG2 was quite reproducible and valid in a large sample of the healthy adults, especially for MVPA. Therefore, it is of good reference value to compare the accuracy of BG2 based on the PA monitoring results.

The results showed that BG2 is capable of accurately monitoring and accumulating time spent in different intensity zones during free-living. The estimation of PA monitoring by BG2 is accurate compared with GT3X+, which proves its appropriateness for research in a real free-living environment. Compared with GT3X+, BG2 estimated lower level of SD and higher level of LPA, whereas MVPA were comparable. Such results may be due to the fact that BG2 is more sensitive to LPA activity in terms of wearing location. In specific, GT3X+ is worn on the hip, the acceleration detected by the device is mainly from the movements of lower quadrant, whereas BG2 is worn on chest, where more slight upper body movement (i.e., sweeping, mopping, washing clothes, upper body resistance training) might be included in LPA (25). So, the magnitude of the measurement bias also varied depending on the PA types (26, 27). In addition, BG2 integrates the HRV data with individual calibrations of HRV responses to free-living physical activities so as to eliminate individual differences in EE caused by physiological differences of participants such as aerobic fitness (28). The difference shown in BG2 measurements may also be caused by HRV correction of ACC counts. The SD time was difficult to be distinguished from LPA when the participants were standing or engaging in some very light activity with GT3X+. In contrast, BG2 can accurately classify SD and LPA time based on the HRV, thus potentially correcting the less accurate estimation of GT3X+. Similarly, the slight measurement difference in MVPA by BG2 may also be related to the correction effect of HRV. As for the comparison of GT3X+ with BG2 in time spent in different intensity zones, the systematic bias between relationships is a negative correlation. That means the lower intensity, the more significant difference between BG2 and GT3X+ measurements, which indicates that BG2 seems to be better corrected when measuring the lower intensity activities.

The results of Bouchard compared with GT3X+ are lower estimated in SD and higher estimated in MVPA, similar to other research. Previous studies have shown that questionnaire-based monitoring tools that the duration and intensity of MVPA recorded by subjects may be higher than objective measures of PA, such as doubly labeled water (DLW) (29, 30). This may be due to differences in understanding and seriousness of the questionnaire among participants. In particular, it was participants' comprehension of different walk speeds in levels 4 and 5 that directly led to the increases in TEE and PAEE. Bouchard is a recall questionnaire in which subjects may make subjective errors when reflecting the intensity and duration of their PA. In addition, as Bouchard uses a fixed interval of 15 min, the total amount of time the subject fills out may differ from the amount of time spent engaging in PA.

In this study, the estimation of Bouchard is the highest in TEE and PAEE, while GT3X+ and BG2 showed no significant difference. Such finding was concordant with previous studies that analyzed the consistency between self-reported levels of PA (similar to Bouchard) and single ACC (21, 31). However, previous studies (32–34) showed high-percentage error and wide limits of agreement in EE estimation using Actigraph when compared with the golden standard tests, while only a few studies showed good validity in predicting EE in controlled treadmill activities or using light intensity stepping exercise (35, 36). Therefore, it remains inconclusive to determine which method was better due to the lack of the gold standard method of EE estimation in the study. It only might be inferred that EE estimated by BG2 is closer to GT3X+.

The BG2 appears to monitor PA under free-living accurately. Despite this, there are still a few limitations: (i) the differences between races are not accounted for in the existing algorithms; (ii) poor contact can cause noise or signal loss, especially during moderate-to-high intensity PA. We also need to handle the poor signal contact of BG2 between the electrode pads and the skin in use; (iii) it was requested that participants remove the equipment while showering and swimming, which might affect measurement results; (iv) BG2 is also susceptible to interference from static electricity in free-living or other metal-based appliances. There also needs to be machine learning or video recognition to verify the activity monitoring using BG2 and the assessment of EE estimate of BG2 using calorimetry in further studies.

In conclusion, by comparing the three methods for the estimation of different PA intensities and EE, BG2 showed comparable results with GT3X+ in both EE and MPVA, and therefore, researchers may use BG2 in future studies for relevant areas. However, there were large discrepancies between SD and LPA prediction. With the potential limitations and drawbacks of using the accelerometer in PA quantification reported by previous studies, it is speculated that a more accurate SD and LPA measurement would be guaranteed when HRV data were combined in BG2. On the other hand, although EE estimation between the three methods was very similar, it only provided an approximate estimation instead of high-precision measurement. Future studies using different populations and the comparison with the gold standard EE test are needed.

## Data availability statement

The original contributions presented in the study are included in the article/supplementary material, further inquiries can be directed to the corresponding author/s.

## Ethics statement

The studies involving human participants were reviewed and approved by the Experimental Ethics Committee approved the study for Sports Science at Beijing Sport University (Registration Number 2022056H). The patients/participants provided their written informed consent to participate in this study.

## Author contributions

Conceptualization: HaocL, QL, YH, DB, HaoyL, and YC. Data curation and writing–original draft: HaocL and QL. Formal analysis and investigation: HaocL, QL, YL, and YW. Writing–review and editing: DB, HaoyL, and YC. All authors contributed to the article and approved the submitted version.

## Funding

This work was supported by the National Key R&D Program of China, Grant Number 2019YFF0301803. The author YH was supported by the Major Project of Beijing Social Science Foundation (20ZDA19).

## Conflict of interest

The authors declare that the research was conducted in the absence of any commercial or financial relationships that could be construed as a potential conflict of interest.

## Publisher's note

All claims expressed in this article are solely those of the authors and do not necessarily represent those of their affiliated organizations, or those of the publisher, the editors and the reviewers. Any product that may be evaluated in this article, or claim that may be made by its manufacturer, is not guaranteed or endorsed by the publisher.
